# Using marine isoscapes to infer movements of oceanic migrants: The case of Bulwer’s petrel, *Bulweria bulwerii*, in the Atlantic Ocean

**DOI:** 10.1371/journal.pone.0198667

**Published:** 2018-06-12

**Authors:** Marta Cruz-Flores, Teresa Militão, Raül Ramos, Jacob González-Solís

**Affiliations:** Institut de Recerca de la Biodiversitat (IRBio) and Departament de Biologia Evolutiva, Ecologia i Ciències Ambientals, Universitat de Barcelona, Barcelona, Spain; Phillip Island Nature Parks, AUSTRALIA

## Abstract

Studying the movements of oceanic migrants has been elusive until the advent of several tracking devices, such as the light-level geolocators. Stable isotope analysis (SIA) offers a complementary approach to infer areas used year-round, but its suitability in oceanic environments remains almost unexplored. To evaluate SIA as a tool for inferring movements of oceanic migrants, we sampled an oceanic seabird, the Bulwer’s petrel, *Bulweria bulwerii*, in four breeding colonies spread along its Atlantic distribution. We first studied the species moulting pattern from 29 corpses collected in the colonies. Secondly, based on this moult knowledge, we selected three feathers from tracked birds to infer their breeding and non-breeding grounds using SIA: the 1^st^ primary (P1), the 8^th^ secondary (S8) and the 6^th^ rectrix (R6) feathers. Birds migrated to two main non-breeding areas, the Central or the South Atlantic Ocean. P1 showed similar isotopic values among petrels from different breeding colonies, suggesting this feather is replaced early in the non-breeding period in a common area used by most birds, the Central Atlantic. S8 and R6 feathers correctly assigned 92% and 81%, respectively, of the birds to their non-breeding areas, suggesting they were replaced late in season, when birds were settled in their main non-breeding grounds. Our results showed that the isotopic baseline levels of the Central and South Atlantic are propagated through the food web until reaching top predators, suggesting these ratios can be used to infer the movement of long-distance migrants among oceanic water masses.

## Introduction

Marine megafauna provide insights into the physical and biological processes occurring in the ocean and are generally considered good indicators of the health and structure of marine ecosystems as well as particularly sensitive to human impacts [1–3]. As such, megafauna, in particular seabirds, are often used to help identifying major hotspots relevant for marine biodiversity and for setting conservation priorities at sea, and therefore there is an increasing need to know their year-round movements. This has boosted a plethora of tracking studies, although most of them have been carried out on terrestrial species or on large pelagic species mainly feeding on neritic areas. In consequence, our knowledge on the use of oligotrophic oceanic areas by oceanic migrants remains relatively scarce.

The proliferation of tracking devices, their increasing autonomy and its continuous miniaturization have enormously increased the number of species that can be tracked [4,5]. However, all devices have some limitations. Some of them need to be recovered, which usually limit their use to study breeders, thus neglecting the movement of non-breeding animals which in long-lived species may conform a large proportion of the population making a different use of the space. Some devices are still relatively expensive, limiting the sample size and the strength of the ecological inferences. In addition, some devices or the way these devices need to be attached, can show detrimental effects [6]. Finally, some species or individuals (e.g. dead animals) may not be possible to track. In this regard, intrinsic biogeochemical markers, such as stable isotopes analysis (SIA), may offer a useful alternative or a complementary approach to infer the areas used year-round by oceanic migrants. Stable isotope values may show geographic gradients at baseline levels [7–9], that are integrated through the food web into their tissues, which ultimately reflect the isotopic values of the area where these tissues were grown [10,11]. Geographical isotopic gradients (i.e. isoscapes) are described in some terrestrial territories (e.g., [12]); nevertheless, they are also now being revealed from the marine environment [8,13]. SIA can overcome some limitations of tracking devices, since tissues can be sampled with minimal disturbance to study any animal of a population (immatures, breeders, non-breeders, sabbaticals, dead or alive individuals) with a single capture, and allows reaching large sample sizes necessary to achieve strong ecological inferences [14]. Feathers of tracked seabirds can be particularly valuable in revealing marine isoscapes and validating the use of stable isotopes as geographic marker of their oceanic movements [15,16], which can later be used to trace the movements of any marine long distance migrant. However, it is crucial to know about its moult chronology and perform SIA in feathers of several birds of known distribution, such as those tracked with GLS (Global Location Sensing) loggers.

In this study, we aim to evaluate the potential of δ^15^N and δ^13^C as geographic intrinsic markers for the study of movements of oceanic migrants. To do so, we used a small oceanic Procellariform breeding along the Macaronesia, the Bulwer’s petrel, *Bulweria bulwerii* (Jardine & Selby, 1828). We hypothesize that major geographic differences in baseline isotopic levels propagate through the food web until reaching top predators, allowing using these differences to study large-scale movements of oceanic migrants. To test this hypothesis, we first assessed the moult chronology of the species using SIA on feathers from bird corpses collected at sampling site. Second, we explored the potential of these isotopes as geographic markers to infer the breeding and non-breeding areas of each bird by determining δ^15^N and δ^13^C values in feathers of birds tracked with geolocators. Finally, we evaluated whether the isotopic differences known to occur lower in the food web (plankton) between the Central and South Atlantic Ocean [9], are consistent with the values found in Bulwer’s petrel feathers.

## Materials and methods

### Study species and sampling strategy

The Bulwer’s petrel is a small pelagic procellariform (75-130g), with a disjointed pan-oceanic distribution in tropical and subtropical waters of the Pacific, Indian and Atlantic Oceans [17,18]. In the latter, it breeds in islets and islands of the Macaronesian archipelagos of Azores, Madeira, Salvages, Canaries and Cape Verde [18]. Adults arrive at the colony from April to May (except in Cima, Cape Verde: late December-January), and leave it from August to October [17,19]. They carry out a partial leapfrog migration, with some individuals from the northern colonies, such as Azores and Canary Islands, migrating further south than individuals from the southern colonies of Cape Verde [19].

We carried out the study on four islets of the Macaronesia: Vila (in Azores Archipelago), Montaña Clara (hereafter M. Clara for brevity, in Canary Islands), Raso, and Cima (both in Cape Verde Archipelago) (Table 1). At each colony, we collected Bulwer’s petrel corpses and sampled a sequence of feathers from them. We also deployed GLS in breeding birds on the four islets, and sampled three specific feathers at the time of GLS recovering.

**Table 1:** Information about the study colonies of Bulwer’s petrel in the Atlantic Ocean.

Islet	Archipelago	Latitude	Longitude	Estimated population size	Breading season	References
Vila	Azores	36.94	-25.17	50 pairs	April-October	[17,19]
M. Clara	Canary Islands	29.29	-13.53	100–130 pairs	April-September	[19,20]
Raso	Cape Verde	16.61	-24.58	Tens of pairs	April-October	[17,19]
Cima	Cape Verde	14.97	-24.64	Tens of pairs	January-August	[17,19]

Geographic position, estimated population size and breeding phenology of Bulwer’s petrel colonies in the Atlantic Ocean included in this study.

### Bird tracking data (GLS)

From 2008 to 2014, we fitted breeding adults with a small combined GLS-immersion logger leg-mounted with a PVC ring (S1 Table). Over the study period, the models we used were Mk13, Mk14, Mk18 and Mk4083 from BAS (British Antarctic Survey, Cambridge, UK), weighing 1.4–2.0 g (1.9–2.7% of a seventy-five-gram Bulwer’s petrel). Overall, we recovered 86 GLS loggers from 65 individuals in the four colonies: 7 GLS loggers from Vila in 2008, 45 from M. Clara (14 from 2011, 13 from 2012, 14 from 2013 and 4 from 2014), 15 from Raso (7 from 2008, 4 from 2009 and 4 from 2010) and 19 from Cima (5 from 2011 and 14 from 2012; S1 Table).

GLS loggers provide two positions per day based on light levels (at local midday and at local midnight), with a mean accuracy of 186 ± 114 km (2° of latitude and longitude [21]). We calculated positions using BASTrack software (BAS). We set a light threshold of 20 and we inspected the integrity of the light curve day-by-day to estimate, when necessary, dawn and dusk times. We excluded 1) transitions where the minimum dark period was less than 4 hours, 2) transitions belonging to the 20 closest days to the equinoxes, when latitude estimation is unreliable, 3) and incubation periods, based on light data recorded by the logger. We applied an iterative forward/backward averaging velocity filter to remove unreliable locations [22], which were those above the 95^th^ percentile of the maximum travel speed of the tracked birds [23].

We defined the non-breeding period as the interval between the date of departure and the date of arrival to the breeding grounds, both determined visually using BASTrack software. We set the departure date as the first day the bird locations were outside the cluster of positions frequented during the breeding period, followed by directed movement away from this area. We determined the arrival date as the first date the bird entered that cluster of positions, preceded by directed movement towards that area. When the date of arrival was impossible to determine visually, we used activity data. Each GLS logger registered saltwater immersion every 3 s and stored the number of immersions in each 10 min period as a value from 0 (continuously dry) to 200 (continuously wet). Thereby, if the bird was in dry mode during all night we inferred it was at the colony, inside the burrow.

### Feathers sampling and stable isotopes analyses (SIA)

To choose the feathers that were moulted within the periods of interest for SIA, it is crucial to know the moulting pattern. Unfortunately, moulting pattern of Bulwer’s petrel is hardly known. The renewal of remiges and rectrices of adult Bulwer’s petrels from most population probably occurs when birds are away from the breeding grounds, from October to April [24,25]. Nevertheless, the moult chronology may differ slightly among populations that have different breeding phenology (Cape Verde vs. more northerly populations [18]). To better understand the moult chronology of the Bulwer’s petrel, we analysed δ^15^N and δ^13^C values on a sequence of feathers from 29 corpses collected at each colony (8 from Vila, 8 from M. Clara, 9 from Raso and 4 from Cima), from 2003 to 2011. From copses, we sampled five primary [the 1^st^ and innermost (P1), 3^rd^ (P3), 5^th^ (P5), 7^th^ (P7) and 10^th^ (P10)], and three secondary [the 1^st^ and outermost (S1), 8^th^ (S8) and 12^th^ (S12)] feathers, whenever possible. To relate feathers isotopic values with breeding and non-breeding areas of each bird, from tracked birds, we sampled one P1, one S8 (from different wings), and one R6 at the time of GLS logger recovery.

We washed feathers in a 0.25 M sodium hydroxide solution, rinsed twice in distilled water to remove any surface contaminant, dried for 24 hours in an oven at 40°C to constant mass, and cut in small pieces to get a homogeneous sample. We placed sub-samples of 0.300 to 0.320 mg (weighed to the nearest 1 μg in a Mettler Toledo MX5) in tin capsules and crimped for combustion for δ^15^N and δ^13^C determination. Isotopic analyses were carried out at the Serveis Científico-Tècnics of the Universitat de Barcelona (Spain) using of a Thermo-Finnigan Flash 1112 (CE Elantech, Lakewood, NJ, USA) elemental analyser coupled to a Delta-C isotope-ratio mass spectrometer via a CONFLOIII interface (Thermo Finnigan MAT, Bremen, Germany). Standards from the International Atomic Energy Agency (IAEA) were used (IAEA N_1_, IAEA N_2_, IAEA NO_3_, IAEA 600, USGS 34 and USGS 40 for N; and IAEA CH_6_, IAEA CH_7_, IAEA 600 and USGS40 for C; S2 Table) and two standard material samples were inserted every 12–16 feather samples to calibrate the system. Stable isotope ratios were expressed in the standard δ-notation relative to Vienna Pee Dee Belemnite (δ^13^C) and atmospheric N_2_ (δ^15^N), according to the following equation: δX = [(R_sample_/R_standard_) − 1], where X (‰) is ^15^N or ^13^C and R are the corresponding ratio ^15^N/^14^N or ^13^C/^12^C related to standard values. Replicate assays of standards material samples indicated standard deviation of maximum ±0.3 and ±0.2 ‰ for δ^15^N and δ^13^C respectively (S2 Table).

### Spatial and statistical analyses

We first assessed the non-breeding area of the last non-breeding period recorded by each GLS logger (in case the GLS logger was deployed more than one year) by performing a 5% kernel density estimates (KDE) constructed with default smoothing parameter *h*, using the package ‘adehabitatHR’ [26] from the R software [27] and then calculating the centroid of those kernels. To perform a partitioning clustering with the non-breeding areas, we applied the partition around medoids (pam) algorithm to an orthometric distance matrix created among all the centroids for the non-breeding areas, and we determined a priori the number of clusters as the number that maximized the overall average silhouette width criterion, as a measure of similarity between an object with its own cluster compared with others [28,29]. To do this, we used the ‘pamk’ function of the R package ‘fpc’ [30].

To compare the δ^15^N and δ^13^C values among breeding and non-breeding areas for each feather (P1, S8 and R6), we performed different tests: one-way ANOVAs and Tukey’s honestly significant difference tests (as post-hoc comparisons) for parametric data, and Kruskal-Wallis tests for non-parametric data.

To check the potential of isotopic values of feathers sampled from the tracked birds to infer their breeding and non-breeding areas, we performed a linear discriminant function analyses (LDA) for each feather (P1, S8 and R6) using ‘linDA’ function from the R package ‘DiscriMiner’ [31]. To test the discriminant efficiency of stable isotopes, we split our dataset in two: the training and the testing datasets including 70% and 30% of the analysed feathers, respectively. To avoid pseudo-replication in the training dataset used to construct the LDA, we randomly selected one feather per bird in the case of animals tracked several years. To test the LDA obtained with the training dataset, we used the rest of the feathers not used in the training dataset, including some feathers from repeated individuals. If the number of feathers in the testing dataset was <30% of the total per colony or per non-breeding area, we randomly selected some feathers from the training data until reaching the 30%. We included breeding and non-breeding areas with the same weight using uninformative priors.

To evaluate the discriminant functions, we explored biplots to compare δ^15^N and δ^13^C values among feathers of tracked birds: P1 (differentiated by colony) versus S8 and R6 (both feathers differentiated by non-breeding areas). In addition, we explored biplots for δ^15^N and δ^13^C for each feather separately (P1, S8 and R6) differentiated by colony of origin and non-breeding areas, and we carried out Standard Bayesian Ellipses using the R package Stable Isotope Bayesian Ellipses in R (‘SIBER’) [32].

Finally, to visualize geographical isotopic gradients of the feathers inferred to be moulted during the non-breeding period based on the results of the Discrimination Function Analyses, i.e. S8 and R6, we created surface interpolation models for δ^15^N and δ^13^C values, using ArcGIS 10.2.2. [33]. We associated the values of the feathers with the position of every centroid for the non-breeding period and we used the Kriging option in Spatial Analyst Tools with Ordinary methods and Spherical semivariogram model. Around every centroid, we limited the interpolation with a mask of 4 degrees using Multiple Ring Buffer option in Analysis Tools. To visualize the results, we used a Mollweide projection background map.

### Ethic statement

All procedures involving animal manipulation were in agreement with the European legislation on the subject. The deployment or recovery of geolocators, and the sampling of feathers, did not take more than 10 minutes by bird, and it did not have visible deleterious effects on study animals. All work in Canary Islands was approved by Consejería de Medio Ambiente del Cabildo de Gran Canaria (research permits 87/2007, 2011/0795), in Azores by the Regional Directorate of the Environment from the Azores (SRAM), and in Cape Verde by Direção-Geral do Ambiente (research permits 01/2009, 02/2010, 01/2011, 01/2012), which began to emit research permits in 2009.

## Results

Isotopic values of δ^15^N of feathers from 29 corpses revealed that variability in P1, P3 and P5 feathers was relatively low for the four colonies, particularly when compared with that in P7, P10, S1, S8 or S12. The same low variability in P1 between colonies was also detected in tracked birds regardless of the non-breeding areas, while the variability increased for the S8 and the R6 and showed differences between non-breeding areas (S1 Fig). Isotopic values of δ^13^C for corpses showed a different pattern, with a higher variability for the birds of Cima regardless of the feather (S1 Fig).

**Fig 1:**
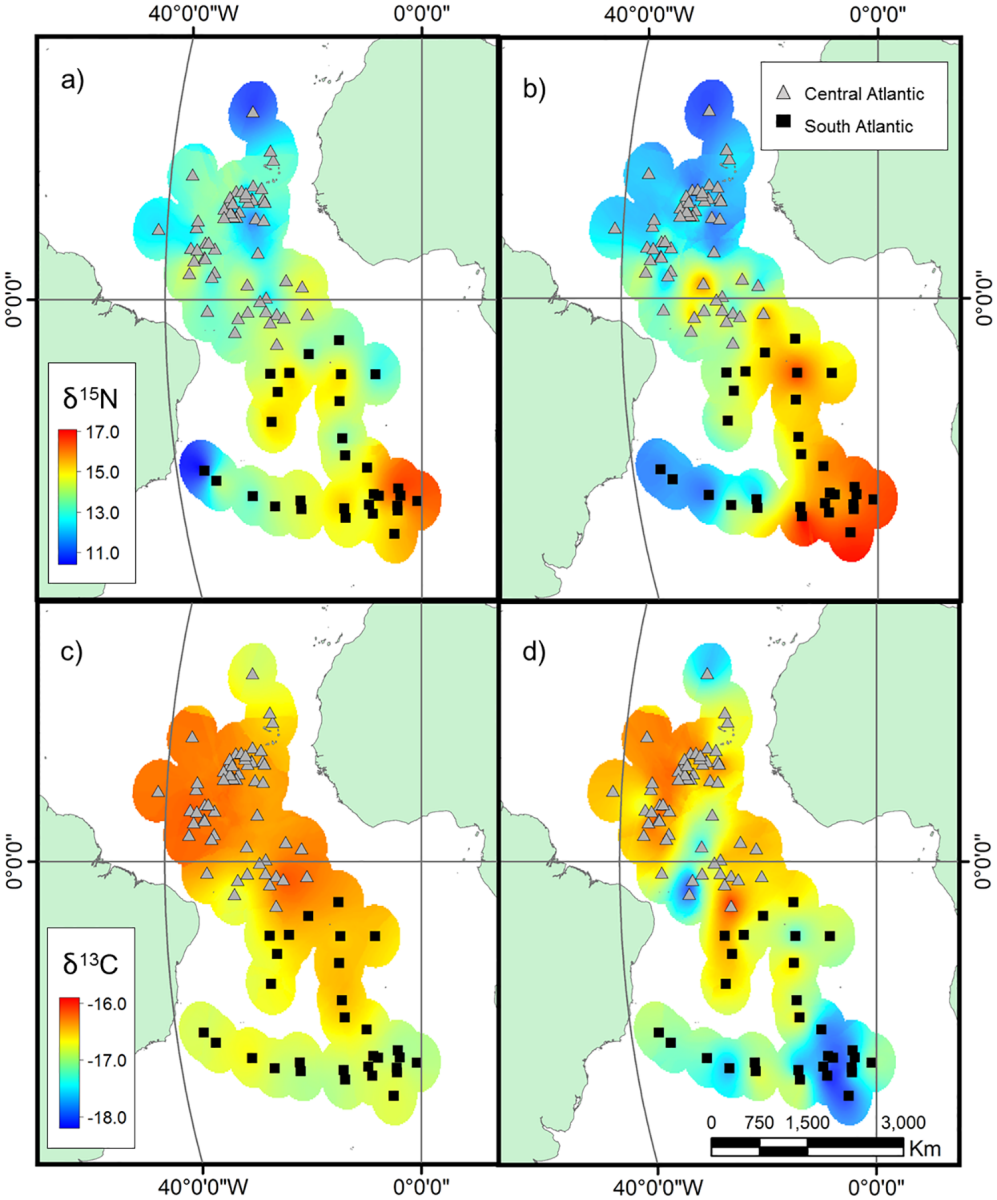
Non-breeding areas and Atlantic surface interpolation models for δ^15^N values of (a) S8 and (b) R6 feathers and δ^13^C values of (c) S8 and (d) R6 feathers. Centroids of the 5% kernel density of the non-breeding distribution of Bulwer’s petrels tracked with GLS loggers (86 trips), and their respective non-breeding areas: the Central Atlantic (grey triangles) and South Atlantic (black squares). Atlantic surface interpolation models for δ^15^N and δ^13^C values of S8 and R6 feathers of Bulwer’s petrels tracked with GLS loggers, and formed with a buffer of 4 degrees around every centroid and using Mollweide projection background map.

The number of clusters that maximized the overall average silhouette width criterion obtained for the centroids of non-breeding areas of the tracked birds was two, corresponding to the Central and South Atlantic, represented in Fig 1.

**Fig 2:**
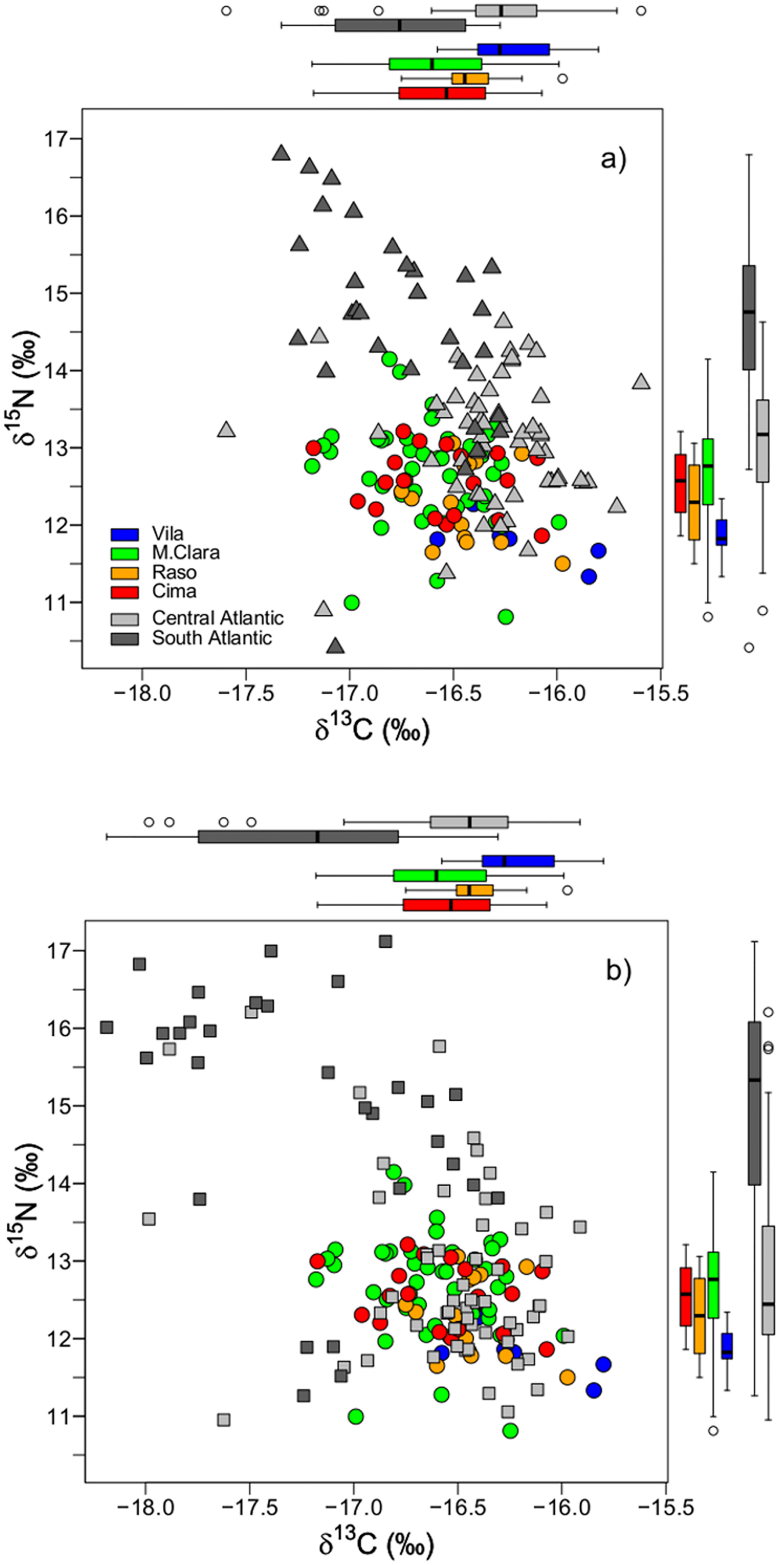
Biplots of δ^15^N and δ^13^C for sampled feathers of tracked animals. Biplots for P1 and S8 (a), and P1 and R6 (b). P1 are depicted in circles and by colony (blue for Vila, n = 7, green for M. Clara, n = 45, orange for Raso, n = 15, and red for Cima, n = 19) and S8 and R6 are depicted with triangles and squares respectively, and by non-breeding areas (grey for Central Atlantic, and dark grey for South Atlantic).

Regarding the differences in δ^15^N and δ^13^C values of P1 feathers among colonies for tracked birds, we found significant differences in both isotopes, being Vila different from M. Clara and Cima, but not from Raso (Table 2). The isotopic values of the P1 from all colonies were similar to those S8 and R6 of birds that spent the non-breeding period in the Central Atlantic (Fig 2). However, there were no significant differences among colonies in S8 feathers for any isotopic ratio. For R6 feathers, we did not find significant differences in δ^15^N values among colonies, but there were significant differences in δ^13^C (Table 2). Regarding the differences for tracked birds between the two non-breeding areas, Central and South Atlantic, δ^15^N and δ^13^C values for the P1 did not differ, but there were significant differences for both isotopes in S8 and R6, being the Central Atlantic more depleted in δ^15^N and more enriched in δ^13^C values than the South Atlantic (Table 2, Fig 2).

**Table 2:** Mean (±SD) isotopic values of sampled feathers by colonies (Vila, M. Clara, Raso and Cima) and non-breeding areas (Central and South Atlantic). Statistical results of either One-way ANOVA or Krystal-Wallis (K-W) with different letters indicating significant differences in post-hoc multiple comparisons.

Feathers	Isotopes	Vila	M. Clara	Raso	Cima	Test for colonies	Central Atlantic	South Atlantic	Test for clusters
P1	δ^15^N	11.9 ± 0.4 a	12.7 ± 0.7 b	12.3 ± 0.5 ab	12.6 ± 0.4 b	F_3,82_ = 4.8, *P*-value = 0.004	12.5 ± 0.6	12.5 ± 0.7	F_1.84_ = 0.2, *P*-value = 0.698
	δ^13^C	-16.2 ± 0.3 a	-16.6 ± 0.3 b	-16.4 ± 0.2 ab	-16.5 ± 0.3 b	F_3,82_ = 5.7, *P*-value = 0.001	-16.5 ± 0.3	-16.5 ± 0.3	F_1.84_ = 0.0, *P*-value = 0.912
S8	δ^15^N	13.8 ± 0.8	13.9 ± 1.5	13.4 ± 0.9	13.1 ± 0.6	F_3,82_ = 2.5, *P*-value = 0.069	13.1 ± 0.8	14.6 ± 1.3	K-W = 30.6, *P*-value < 0.001
	δ^13^C	-16.4 ± 0.4	-16.6 ± 0.5	-16.3 ± 0.2	-16.3 ± 0.2	K-W = 7.6, *P*-value = 0.056	-16.3 ± 0.3	-16.8 ± 0.3	K-W = 28.5, *P*-value < 0.001
R6	δ^15^N	13.8 ± 2.3	13.9 ± 1.8	13.2 ± 1.3	13.0 ± 1.2	K-W = 3.8, *P*-value = 0.283	12.8 ± 1.2	15.0 ± 1.6	K-W = 25.9, *P*-value < 0.001
	δ^13^C	-16.7 ± 0.4 ab	-17.0 ± 0.7 b	-16.5 ± 0.3 a	-16.5 ± 0.3 a	K-W = 9.7, *P*-value = 0.022	-16.5 ± 0.4	-17.2 ± 0.5	K-W = 30.0, *P*-value < 0.001

Based on the δ^15^N and δ^13^C values, all feathers from tracked birds showed low values of correct assignment to the respective breeding colonies (Table 3). In contrast, S8 and the R6 reached 92% and 81%, respectively, of correct assignment to the corresponding non-breeding areas of the tracked birds (Table 3). Discriminant functions for the non-breeding grounds for Bulwer’s petrel are shown in Table 3.

**Table 3:** Correct classification rates (%) for colonies and non-breeding areas by feather (A), and discriminant functions using SIA (B).

**A**								
	Sample sizes		P1 correct classification (%)		S8 correct classification (%)		R6 correct classification (%)	
	Training	Testing	Training	Testing	Training	Testing	Training	Testing
**Breeding colonies**:								
Vila	5	2	40.0	50.0	20.0	0.0	0.0	0.0
M. Clara	31	14	51.6	57.1	35.5	21.4	48.4	50.0
Raso	10	5	60.0	0.0	20.0	40.0	50.0	40.0
Cima	12	5	76.9	33.3	38.5	50.0	30.8	33.3
Total	**58**	**26**	**42.4**	**40.7**	**32.2**	**29.6**	**40.7**	**40.7**
**Non-breeding areas**:								
Central Atlantic	38	16	51.3	47.1	87.2	94.1	89.7	82.4
South Atlantic	21	9	61.9	77.8	76.2	88.9	66.7	77.8
Total	**59**	**25**	**55.0**	**57.7**	**83.3**	**92.3**	**81.7**	**80.8**
**B**								
**Discriminant functions**:	Central Atlantic				South Atlantic			
8^th^ secondary (S8)	5.8×*δ*^15^*N* − 116.2×*δ*^13^*C* − 983.7				7.0×*δ*^15^*N* − 118.8×*δ*^13^*C* − 1043.9			
6^th^ rectrix (R6)	1.3×*δ*^15^*N* − 79.0×*δ*^13^*C* − 660.9				2.3×*δ*^15^*N* − 82.2×*δ*^13^*C* − 727.1			

Correct classification rates (%) obtained using stable isotope analysis (δ^15^N and δ^13^C values) for P1, S8 and R6 of Bulwer’s petrel tracked with geolocation, and validation of the discriminant analyses using the testing data. Discriminant functions obtained using δ^15^N and δ^13^C values of S8 and R6 to infer the non-breeding areas of Bulwer’s petrel. For each feather, the higher result (for the Central or South Atlantic) indicates the belonging of the bird to that cluster.

Standard Bayesian Ellipses performed separately by feather type, colony and non-breeding area for tracked birds, showed P1 isotopic values largely overlap among colonies and regardless of the non-breeding areas visited by the birds, except for the birds from Vila that spent the non-breeding period in the South Atlantic, which showed enriched δ^13^C values. Isotopic values of S8 segregated between the two non-breeding areas for birds breeding in M. Clara and Vila, while overlapped extensively for all birds breeding in Raso and Cima. Isotopic values of R6 segregated between non-breeding areas for birds breeding in all colonies, even though segregation was more pronounced for birds breeding in Vila and Cima than for those breeding in M. Clara and Raso (S2 Fig).

Surface interpolation models for S8 and R6 pointed out geographic differences in the isotopic values between the two non-breeding areas. For both feathers, we found more enriched δ^15^N and depleted δ^13^C values for birds wintering in the South Atlantic than for those wintering in the Central Atlantic, with a spot with lower values of δ^15^N in the South Atlantic closer to the coastline of Brazil. For both isotopes, the gradient was more noticeable for the R6 than for the S8 (Fig 1).

## Discussion

In this study, by combining SIA of different feathers with the migratory tracks of a small oceanic seabird instrumented with geolocators, we showed δ^15^N and δ^13^C values in the feathers grown in Central and South Atlantic regions differ. More specifically, we showed these differences are consistent with those known to occur lower in the food web and can be effectively used to infer the non-breeding areas of the Bulwer’s petrel in the Central and South Atlantic Ocean. This result agrees with our hypothesis, showing differences in the basal isotopic values propagate through the food web until reaching top predators, therefore indicating that it is possible to use SIA as intrinsic marker to trace large-scale movement of long-distance migrants among vast oceanic waters.

First, our isotopic results on several feathers of corpses collected at different colony sites unravelled moulting pattern of remiges for this oceanic species. In other sympatric Procellariiformes, such as Cory’s shearwater, moulting starts at the end of breeding, while still rearing the chick [34,35]. If this was the case for Bulwer’s petrel, and based on plankton marine isoscapes defined along the Atlantic [9,13], we would expect to detect some differences in the isotopic values of P1 feathers among the four studied populations, but this was not the case. The similarity of the isotopic signal from P1 to P5 among birds from different populations indicate the moult of the innermost primary feathers occurs after the breeding period, as previously suggested by [25]. In particular, δ^15^N values of P1, P3 and P5 from corpses from the different populations showed low variability when compared with other feathers (e.g., P10 and S8), also in line with that registered in P1 feathers of the tracked birds (S1 Fig). Indeed, isotopic values of P1 feathers of most populations largely overlapped with those of S8 and R6 feathers from birds that spent the entire non-breeding period on the Central Atlantic, suggesting all birds from different population replaced these feathers in this area. All Bulwer’s petrels visited this area at the onset of the non-breeding period and, even those that headed to the South Atlantic in a later stage, first spent some time on it. In addition, isotopic values for birds from Vila, which spent the non-breeding period in the South Atlantic, showed P1 more enriched in carbon than birds from other archipelagos, suggesting that birds from this islet may moult the P1 in a more neritic area [36]. For all the colonies, outermost primary (i.e., P7 and P10) and secondary (S1, S8 and S12) feathers sampled from corpses, as well as those S8 and R6 feathers from tracked birds, showed large variability in their δ^15^N values, particularly when compared to the isotopic variability of P1 feathers. A large variability in the isotopic values of these feathers pointed out these were moulted in different areas with distinct isotopic baselines. Indeed, we found significant differences in the isotopic values of S8 and R6 feathers of tracked birds that wintered in the Central and the South Atlantic Ocean, suggesting these feathers are moulted later in the non-breeding season, when birds are already settled in their main wintering area.

The reduction of the non-breeding areas of tracked birds to its centroid, the limited accuracy of the GLS positions, the difficulty in identifying the staging areas of the Bulwer’s petrels over their migratory period, the annual variation in the baseline isotopic values and the variability in the moulting patterns among birds and among populations, may obscure or dilute the relationship between the geographic position of the wintering areas, as indicated by the geolocators, and the isotopic values of the feathers inferred to be moulted in these areas. Despite of all these potentially confounding factors, the isotopic differences of S8 and R6 feathers between tracked birds that wintered in the Central and the South Atlantic Ocean allowed us to correctly assign 92% and 81% of the birds, respectively, to their putative non-breeding areas. Indeed, the geographic isotopic differences found in Bulwer’s petrels mirror the marine isoscapes for plankton [8,13]. That is, in general both Bulwer’s petrel feathers and plankton showed a gradient with higher δ^15^N and lower δ^13^C values in the South than in the Central Atlantic, with an area with lower δ^15^N values in the South Atlantic closer to the coastline of Brazil. This result indicate baseline isotopic differences between these two areas are propagated through the food web and can be used to study the movements of top predators in oceanic waters.

Our study underline the usefulness of multidisciplinary studies combining SIA and tracking data to bring new insights into the migratory ecology and conservation biology. Firstly, we pointed out how one might unravel unknown moulting patterns of seabird species that are not accessible to researchers during most part of their annual cycle. Secondly, we also described discriminant functions that allow assigning the non-breeding area of any Bulwer’s petrel with a good accuracy, which may also be used for other related species feeding on similar prey [37] but more difficult to track using devices, such as storm-petrels (*Hydrobates* spp). Thirdly, we revealed that isotopic baseline differences in oceanic environments are propagated to top predators, allowing us to trace large-scale movements of long-distance migrants by sampling specific tissues (i.e., feathers), opening new opportunities to study the movements of individuals or species that for a number of logistic, economical or ethical reasons cannot be tracked using electronic devices.

## Supporting information

S1 Table Total number of GLS loggers deployed and recovered by colony, and number of GLS loggers included in the present study. Only GLS loggers with data for the last non-breeding period before recovering were included in this study, in order to relate the non-breeding areas with the feathers moulted during the non-breeding period and sampled at GLS logger recovering.
(DOCX)

S2 Table Accepted values of the standard material used in the stable isotopic analysis performed in this study, mean measured (±standard deviation) in the samples of standards materials used, minimum and maximum values for all runs, and number of samples (n). (DOCX)

S1 Fig Boxplots of δ^15^N (a) and δ^13^C (b) values for the 1^st^ (P1), 3^rd^ (P3), 5^th^ (P5), 7^th^ (P7) and 10^th^ (P10) primary feathers, the 1^st^ (S1), 12^th^ (S12) and 8^th^ (S8) secondary feathers of Bulwer’s petrels found dead at each colony: Vila (in blue, n = 8), M. Clara (green, n = 8), Raso (orange, n = 9) and Cima (red, n = 4). The 6^th^ rectrix (R6) of corpses are not represented here because the most of them did not have the tail. Also values for δ^15^N and δ^13^C are shown for the P1, S8 and R6 of alive tracked birds that spent the non-breeding period in Central or South Atlantic by colony.
(TIF)

S2 Fig Biplots of δ^15^N and δ^13^C values for P1 (a), S8 (b) and R6 (c) feathers sampled from tracked individuals of Bulwer’s petrel. In each plot, we show isotopic values and Standard Bayesian Ellipses by non-breeding areas (depicted in triangles and discontinuous contours for birds wintering in the Central Atlantic, and in circles and continuous contours for birds wintering in the South Atlantic) and colony (Vila in blue (n = 3/4 for Central/South Atlantic), M. Clara in green (n = 25/20), Raso in orange (n = 12/3) and Cima in red tones (n = 16/3)).
(TIF)

S1 File Isotopic data of corpses and tracked Bulwer’s petrels included in the article. (DOCX)
