# Correction: Using marine isoscapes to infer movements of oceanic migrants: The case of Bulwer’s petrel, *Bulweria bulwerii*, in the Atlantic Ocean

**DOI:** 10.1371/journal.pone.0213565

**Published:** 2019-03-05

**Authors:** The PLOS ONE Staff

The Table 3 caption incorrectly appears as the fifth paragraph of the Results section. Please see the complete, correct Table 3 and Table 3 caption here. The publisher apologizes for the error.

**Table 3:** Correct classification rates (%) for colonies and non-breeding areas by feather, and discriminant functions using SIA.

			P1 correct		S8 correct		R6 correct	
	Sample sizes		classification (%)		classification (%)		classification (%)	
	Training	Testing	Training	Testing	Training	Testing	Training	Testing
**Breeding colonies:**								
Vila	5	2	40.0	50.0	20.0	0.0	0.0	0.0
M. Clara	31	14	51.6	57.1	35.5	21.4	48.4	50.0
Raso	10	5	60.0	0.0	20.0	40.0	50.0	40.0
Cima	12	5	76.9	33.3	38.5	50.0	30.8	33.3
Total	**58**	**26**	**42.4**	**40.7**	**32.2**	**29.6**	**40.7**	**40.7**
**Non-breeding areas**								
Central Atlantic	38	16	51.3	47.1	87.2	94.1	89.7	82.4
South Atlantic	21	9	61.9	77.8	76.2	88.9	66.7	77.8
Total	**59**	**25**	**55.0**	**57.7**	**83.3**	**92.3**	**81.7**	**80.8**
**Discriminant functions:**	Central Atlantic				South Atlantic			
8^th^ secondary (S8)	5.8×*δ*^15^ *N* − 116.2×*δ*^13^ *C* − 983.7				7.0×*δ*^15^ *N* − 118.8×*δ*^13^ *C* − 1043.9			
6^th^ rectrix (R6)	1.3×*δ*^15^ *N* − 79.0×*δ*^13^ *C* − 660.9				2.3×*δ*^15^ *N* − 82.2×*δ*^13^ *C* − 727.1			

Correct classification rates (%) obtained using stable isotope analysis (δ^15^N and δ^13^C values) for P1, S8 and R6 of Bulwer’s petrel tracked with geolocation, and validation of the discriminant analyses using the testing data. Discriminant functions obtained using δ^15^N and δ^13^C values of S8 and R6 to infer the non-breeding areas of Bulwer’s petrel. For each feather, the higher result (for the Central or South Atlantic) indicates the belonging of the bird to that cluster.
