# Supplementary material for: Using marine isoscapes to infer movements of oceanic migrants: The case of Bulwer’s petrel, Bulweria bulwerii, in the Atlantic Ocean
Source: PLoS One. 2018 Jun 12;13(6):e0198667. doi: 10.1371/journal.pone.0198667 (PMC5997309; doi:10.1371/journal.pone.0198667)
Supplement: S1 Table — Only GLS loggers with data for the last non-breeding period before recovering were included in this study, in order to relate the non-breeding areas with the feathers moulted during the non-breeding period and sampled at GLS logger recovering. (DOCX) [file pone.0198667.s001.docx]

**S1 Table.** Total number of GLS logger deployed and recovered by colony, and number of GLS loggers included in the present study. Only GLS loggers with data for the last non-breeding period before recovering were included in this study, in order to relate the non-breeding areas with the feathers moulted during the non-breeding period and sampled at GLS logger recovering.

| Colony |  | Deployed GLS |  | Recovered GLS |  | GLS included in the study |
| --- | --- | --- | --- | --- | --- | --- |
| Vila |  | 13 (2007) |  | 7 (2008) |  | 7 |
| M. Clara |  | 106 (2010-2013) |  | 68 (2011-2014) |  | 45 |
| Raso |  | 35 (2007-2009) |  | 23 (2008-2010) |  | 15 |
| Cima |  | 40 (2010-2011) |  | 20 (2011-2012) |  | 19 |
