# Supplementary material for: Using marine isoscapes to infer movements of oceanic migrants: The case of Bulwer’s petrel, Bulweria bulwerii, in the Atlantic Ocean
Source: PLoS One. 2018 Jun 12;13(6):e0198667. doi: 10.1371/journal.pone.0198667 (PMC5997309; doi:10.1371/journal.pone.0198667)
Supplement: S2 Table — (DOCX) [file pone.0198667.s002.docx]

**S2 Table.** Accepted values of the standard material used in the stable isotopic analysis performed in this study, mean measured (±standard deviation) in the samples of standards materials used, minimum and maximum values for all runs, and number of samples (n).

| Standard material |  | δ^15^N_AIR_(‰ ) | | | | |  | δ^13^C_VPDB_(‰ ) | | | | |  |  |
| --- | --- | --- | --- | --- | --- | --- | --- | --- | --- | --- | --- | --- | --- | --- |
|  |  | Accepted values |  | Measured values | | |  | Accepted values |  | Measured values | | |  | References of the accepted values |
|  |  | Mean ± SD |  | Mean of all runs ± SD | Minimum-maximum | n |  | Mean ± SD |  | Mean of all runs ± SD | Minimum-maximum | n |  |  |
| IAEA N1 |  | 0.4 ± 0.1 |  | 0.5 ± 0.3 | -0.2 to 1.1 | 42 |  |  |  |  |  |  |  | [1] |
| IAEA N2 |  | 20.4 ± 0.1 |  | 20.3 ± 0.2 | 19.7 to 20.6 | 42 |  |  |  |  |  |  |  | [2] |
| IAEA NO_3_ |  | 4.7 ± 0.1 |  | 4.6 ± 0.2 | 4.0 to 4.9 | 20 |  |  |  |  |  |  |  | [1] |
| USG 34 |  | -1.8 ± 0.2 |  | -1.8 ± 0.3 | -2.6 to -1.3 | 25 |  |  |  |  |  |  |  | [3] |
| USGS 40 |  | -4.5 ± 0.1 |  | -4.6 ± 0.1 | -4.9 to -4.3 | 48 |  | -26.2 ± 0.1 |  | -26.4 ± 0.1 | -26.6 to -26.2 | 47 |  | [4] |
| IAEA 600 |  | 1.0 ± 0.2 |  | 1.0 ± 0.2 | 0.6 to 1.4 | 34 |  | -27.8 ± 0.0 |  | -27.7 ± 0.2 | -27.9 to -26.8 | 34 |  | [5] |
| IAEA CH6 |  |  |  |  |  |  |  | -10.5 ± 0.0 |  | -10.4 ± 0.1 | -10.7 to -10.3 | 46 |  | [5] |
| IAEA CH7 |  |  |  |  |  |  |  | -32.2 ± 0.1 |  | -32.1 ± 0.1 | -32.3 to -32.0 | 43 |  | [5] |

**References**

1. Böhlke JK, Coplen TB. Interlaboratory comparison of reference materials for nitrogen-isotope-ratio measurements. Reference and intercomparison materials for stable isotopes of light elements. Viena: Proceedings of a consultants meeting - IAEA; 1993. pp. 51–66.

2. Braak CJFT, Smilauer P. CANOCO Reference Manual and user’s guide to Canoco for Windows: Software for Canonical Community Ordination (version 4). Ithaca, NY: Microcomputer Power; 1998. p.

3. Böhlke JK, Mroczkowski SJ, Coplen TB. Oxygen isotopes in nitrate: new reference materials for ^18^O:^17^O:^16^O measurements and observations on nitrate-water equilibration. Rapid Commun mass Spectrom RCM, 2003. 2003;17: 1835–46. doi:10.1002/rcm.1123

4. Qi H, Coplen TB, Gelimann H, Brand WA, Böhlke JK. Two new organic reference materials for δ^13^C and δ^15^N measurements and new value for the δ^13^C of NBS 22 oil. Rapid Commun Mass Spectrom. 2003;17: 2483–2487. Available: http://nucleus.iaea.org/rpst/ReferenceProducts/ReferenceMaterials/Stable_Isotopes/15N14N/USGS34.htm

5. Coplen TB, Brand WA, Gehre M, Gro M, Meijer HAJ, Toman B, et al. New guidelines for δ^13^C measurements. Anal Chem. 2006;78: 2439–2441.
