# Supplementary figures and images for: Using marine isoscapes to infer movements of oceanic migrants: The case of Bulwer’s petrel, Bulweria bulwerii, in the Atlantic Ocean
Source: PLoS One. 2018 Jun 12;13(6):e0198667. doi: 10.1371/journal.pone.0198667 (PMC5997309; doi:10.1371/journal.pone.0198667)

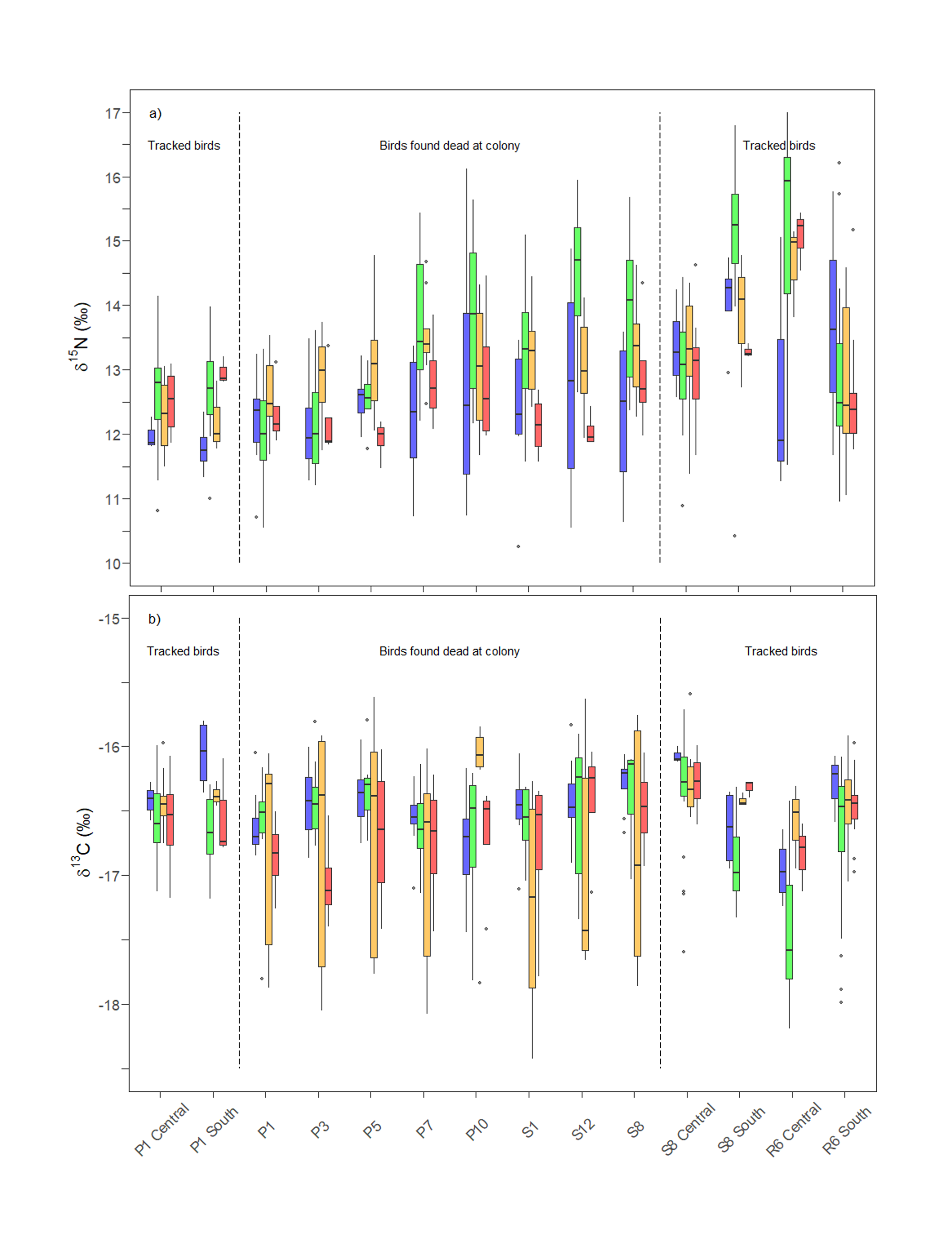

Supplement: S1 Fig — The 6th rectrix (R6) of corpses are not represented here because the most of them did not have the tail. Also values for δ15N and δ13C are shown for the P1, S8 and R6 of alive tracked birds that spent the non-breeding period in Central or South Atlantic by colony. (TIF) [file pone.0198667.s003.tif]

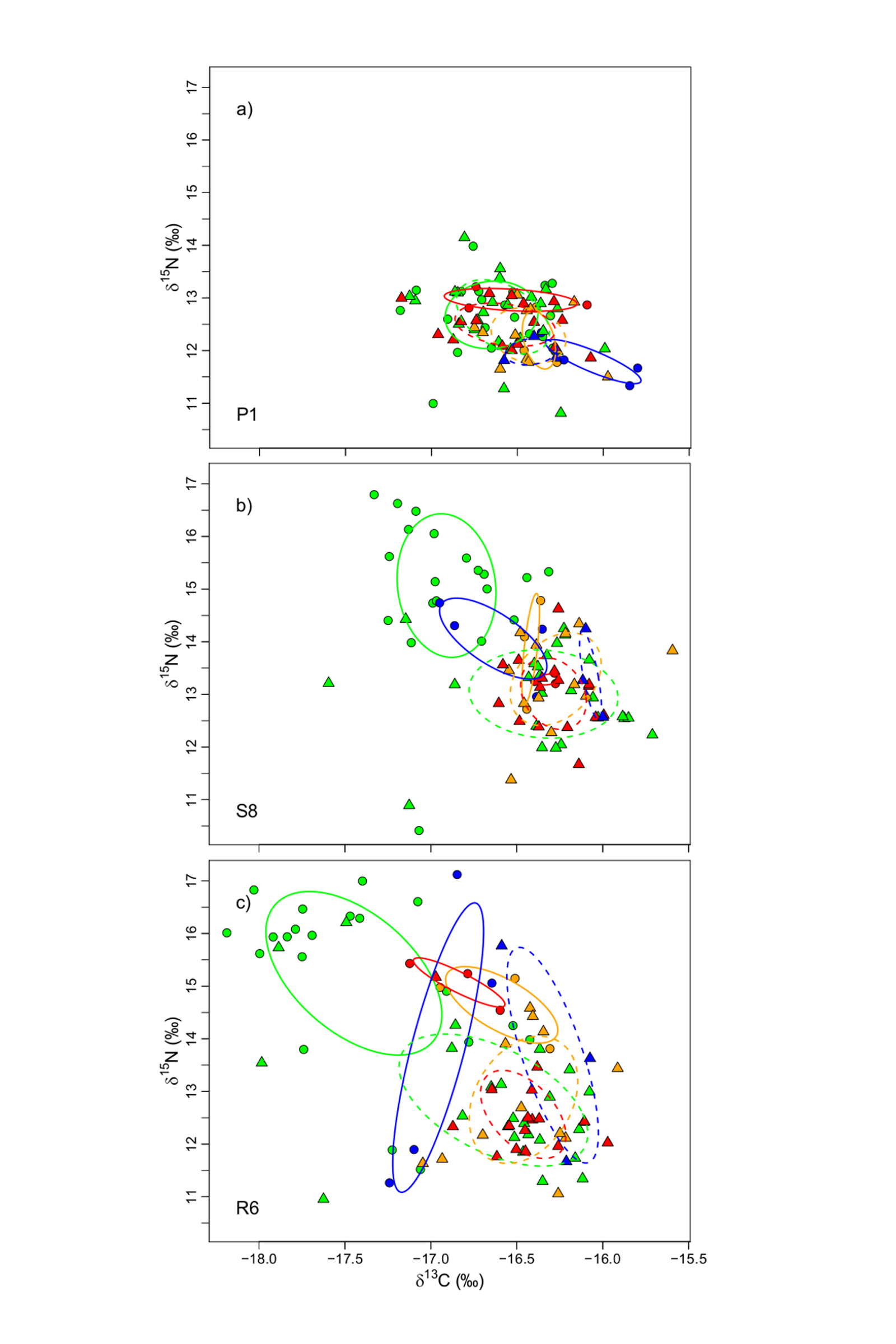

Supplement: S2 Fig — In each plot, we show isotopic values and Standard Bayesian Ellipses by non-breeding areas (depicted in triangles and discontinuous contours for birds wintering in the Central Atlantic, and in circles and continuous contours for birds wintering in the South Atlantic) and colony (Vila in blue (n = 3/4 for Central/South Atlantic), M. Clara in green (n = 25/20), Raso in orange (n = 12/3) and Cima in red tones (n = 16/3)). (TIF) [file pone.0198667.s004.tif]
