# Supplementary material for: Using marine isoscapes to infer movements of oceanic migrants: The case of Bulwer’s petrel, Bulweria bulwerii, in the Atlantic Ocean
Source: PLoS One. 2018 Jun 12;13(6):e0198667. doi: 10.1371/journal.pone.0198667 (PMC5997309; doi:10.1371/journal.pone.0198667)
Supplement: S1 File — (DOCX) [file pone.0198667.s005.docx]

**S1 File.** Isotopic data of corpses and tracked Bulwer’s petrels included in the article.

**Table A.** Isotopic data of a sequence of feathers from Bulwer’s petrel corpses.

| **Breeding area** | | | **Feather** | | **δ^15^N** | | **δ ^13^C** | **Breeding area** | | **Feather** | | **δ^15^N** | **δ ^13^C** |
| --- | --- | --- | --- | --- | --- | --- | --- | --- | --- | --- | --- | --- | --- |
| **Population** | **Islet** |  | |  | |  | | **Population** | **Islet** |  |  | |  |
| Azores | Vila | P1 | | 11.676 | | -16.742 | | Canary | M.Clara | S1 | 13.146 | | -16.311 |
| Azores | Vila | P1 | | 12.349 | | -16.376 | | Canary | M.Clara | S8 | 12.373 | | -16.099 |
| Azores | Vila | P1 | | 12.813 | | -16.737 | | Canary | M.Clara | S8 | 15.680 | | -17.028 |
| Azores | Vila | P1 | | 12.400 | | -16.667 | | Canary | M.Clara | S8 | 12.892 | | -16.106 |
| Azores | Vila | P1 | | 12.463 | | -16.848 | | Canary | M.Clara | S8 | 14.703 | | -16.525 |
| Azores | Vila | P1 | | 11.943 | | -16.792 | | Canary | M.Clara | S8 | 14.084 | | -16.136 |
| Azores | Vila | P1 | | 13.247 | | -16.612 | | Canary | M.Clara | S12 | 15.937 | | -17.338 |
| Azores | Vila | P1 | | 10.705 | | -16.047 | | Canary | M.Clara | S12 | 14.728 | | -16.019 |
| Azores | Vila | P3 | | 11.682 | | -16.512 | | Canary | M.Clara | S12 | 14.027 | | -16.236 |
| Azores | Vila | P3 | | 12.853 | | -16.337 | | Canary | M.Clara | S12 | 14.701 | | -17.089 |
| Azores | Vila | P3 | | 12.251 | | -16.081 | | Canary | M.Clara | S12 | 12.645 | | -15.904 |
| Azores | Vila | P3 | | 11.434 | | -16.005 | | Canary | M.Clara | S12 | 15.682 | | -16.887 |
| Azores | Vila | P3 | | 11.834 | | -16.864 | | Canary | M.Clara | S12 | 13.653 | | -16.152 |
| Azores | Vila | P3 | | 12.053 | | -16.635 | | C.Verde | Raso | P1 | 12.272 | | -16.154 |
| Azores | Vila | P3 | | 13.484 | | -16.670 | | C.Verde | Raso | P1 | 12.510 | | -18.976 |
| Azores | Vila | P3 | | 11.285 | | -16.294 | | C.Verde | Raso | P1 | 13.339 | | -17.872 |
| Azores | Vila | P5 | | 12.583 | | -16.494 | | C.Verde | Raso | P1 | 13.540 | | -17.663 |
| Azores | Vila | P5 | | 13.224 | | -16.338 | | C.Verde | Raso | P1 | 12.284 | | -16.276 |
| Azores | Vila | P5 | | 12.644 | | -15.948 | | C.Verde | Raso | P1 | 11.686 | | -16.291 |
| Azores | Vila | P5 | | 11.957 | | -16.061 | | C.Verde | Raso | P1 | 12.979 | | -17.416 |
| Azores | Vila | P5 | | 12.320 | | -16.689 | | C.Verde | Raso | P1 | 12.437 | | -16.055 |
| Azores | Vila | P5 | | 12.681 | | -16.326 | | C.Verde | Raso | P3 | 12.996 | | -15.991 |
| Azores | Vila | P5 | | 12.740 | | -16.754 | | C.Verde | Raso | P3 | 11.753 | | -15.913 |
| Azores | Vila | P5 | | 12.340 | | -16.381 | | C.Verde | Raso | P3 | 12.553 | | -18.695 |
| Azores | Vila | P7 | | 13.322 | | -16.543 | | C.Verde | Raso | P3 | 13.356 | | -17.788 |
| Azores | Vila | P7 | | 10.718 | | -16.232 | | C.Verde | Raso | P3 | 13.393 | | -17.684 |
| Azores | Vila | P7 | | 11.804 | | -16.491 | | C.Verde | Raso | P3 | 13.744 | | -15.930 |
| Azores | Vila | P7 | | 12.005 | | -16.335 | | C.Verde | Raso | P3 | 12.490 | | -16.765 |
| Azores | Vila | P7 | | 13.366 | | -16.569 | | C.Verde | Raso | P3 | 12.395 | | -15.968 |
| Azores | Vila | P7 | | 13.056 | | -16.548 | | C.Verde | Raso | P3 | 13.042 | | -18.051 |
| Azores | Vila | P7 | | 12.672 | | -16.694 | | C.Verde | Raso | P5 | 13.694 | | -16.434 |
| Azores | Vila | P7 | | 11.144 | | -17.097 | | C.Verde | Raso | P5 | 12.245 | | -15.618 |
| Azores | Vila | P10 | | 13.827 | | -16.592 | | C.Verde | Raso | P5 | 12.525 | | -18.878 |
| Azores | Vila | P10 | | 10.978 | | -16.740 | | C.Verde | Raso | P5 | 13.222 | | -17.767 |
| Azores | Vila | P10 | | 11.511 | | -17.159 | | C.Verde | Raso | P5 | 13.456 | | -17.616 |
| Azores | Vila | P10 | | 12.215 | | -16.169 | | C.Verde | Raso | P5 | 14.771 | | -16.331 |
| Azores | Vila | P10 | | 14.006 | | -16.474 | | C.Verde | Raso | P5 | 12.053 | | -16.075 |
| Azores | Vila | P10 | | 12.676 | | -17.439 | | C.Verde | Raso | P5 | 12.549 | | -15.939 |
| Azores | Vila | P10 | | 16.120 | | -16.666 | | C.Verde | Raso | P5 | 13.097 | | -17.694 |
| Azores | Vila | P10 | | 10.733 | | -16.938 | | C.Verde | Raso | P7 | 13.274 | | -16.364 |
| Azores | Vila | S1 | | 13.456 | | -16.483 | | C.Verde | Raso | P7 | 13.628 | | -16.588 |
| Azores | Vila | S1 | | 12.022 | | -16.420 | | C.Verde | Raso | P7 | 13.401 | | -18.073 |
| Azores | Vila | S1 | | 12.160 | | -16.540 | | C.Verde | Raso | P7 | 13.341 | | -17.733 |
| Azores | Vila | S1 | | 11.964 | | -16.052 | | C.Verde | Raso | P7 | 13.638 | | -17.628 |
| Azores | Vila | S1 | | 13.348 | | -16.614 | | C.Verde | Raso | P7 | 14.674 | | -16.551 |
| Azores | Vila | S1 | | 13.105 | | -16.376 | | C.Verde | Raso | P7 | 12.475 | | -16.014 |
| Azores | Vila | S1 | | 12.442 | | -16.193 | | C.Verde | Raso | P7 | 14.351 | | -16.078 |
| Azores | Vila | S1 | | 10.259 | | -17.103 | | C.Verde | Raso | P7 | 13.072 | | -17.538 |
| Azores | Vila | S8 | | 13.424 | | -16.158 | | C.Verde | Raso | P10 | 11.677 | | -15.887 |
| Azores | Vila | S8 | | 10.631 | | -16.181 | | C.Verde | Raso | P10 | 13.564 | | -16.043 |
| Azores | Vila | S8 | | 11.540 | | -16.249 | | C.Verde | Raso | P10 | 13.978 | | -15.843 |
| Azores | Vila | S8 | | 11.930 | | -16.060 | | C.Verde | Raso | P10 | 12.114 | | -16.179 |
| Azores | Vila | S8 | | 13.580 | | -16.560 | | C.Verde | Raso | P10 | 14.322 | | -16.089 |
| Azores | Vila | S8 | | 13.256 | | -16.225 | | C.Verde | Raso | P10 | 12.553 | | -17.834 |
| Azores | Vila | S8 | | 13.100 | | -16.192 | | C.Verde | Raso | S1 | 13.664 | | -16.458 |
| Azores | Vila | S8 | | 11.032 | | -16.666 | | C.Verde | Raso | S1 | 12.426 | | -18.510 |
| Azores | Vila | S12 | | 13.854 | | -16.570 | | C.Verde | Raso | S1 | 13.294 | | -17.764 |
| Azores | Vila | S12 | | 11.038 | | -16.346 | | C.Verde | Raso | S1 | 13.541 | | -17.911 |
| Azores | Vila | S12 | | 11.619 | | -16.544 | | C.Verde | Raso | S1 | 12.831 | | -16.272 |
| Azores | Vila | S12 | | 12.458 | | -15.833 | | C.Verde | Raso | S1 | 14.451 | | -16.570 |
| Azores | Vila | S12 | | 14.627 | | -16.112 | | C.Verde | Raso | S1 | 12.559 | | -18.422 |
| Azores | Vila | S12 | | 13.195 | | -16.517 | | C.Verde | Raso | S8 | 12.754 | | -17.863 |
| Azores | Vila | S12 | | 14.876 | | -16.434 | | C.Verde | Raso | S8 | 13.343 | | -17.620 |
| Azores | Vila | S12 | | 10.542 | | -16.905 | | C.Verde | Raso | S8 | 13.425 | | -17.644 |
| Canary | M.Clara | P1 | | 11.496 | | -16.160 | | C.Verde | Raso | S8 | 14.625 | | -15.884 |
| Canary | M.Clara | P1 | | 13.317 | | -17.801 | | C.Verde | Raso | S8 | 12.672 | | -17.427 |
| Canary | M.Clara | P1 | | 12.007 | | -16.720 | | C.Verde | Raso | S8 | 13.399 | | -16.419 |
| Canary | M.Clara | P1 | | 10.539 | | -16.616 | | C.Verde | Raso | S8 | 12.264 | | -15.856 |
| Canary | M.Clara | P1 | | 11.704 | | -16.511 | | C.Verde | Raso | S8 | 14.552 | | -15.757 |
| Canary | M.Clara | P1 | | 12.514 | | -16.419 | | C.Verde | Raso | S12 | 11.933 | | -15.630 |
| Canary | M.Clara | P1 | | 12.540 | | -16.444 | | C.Verde | Raso | S12 | 12.660 | | -17.475 |
| Canary | M.Clara | P3 | | 11.494 | | -15.807 | | C.Verde | Raso | S12 | 13.305 | | -17.617 |
| Canary | M.Clara | P3 | | 13.607 | | -16.769 | | C.Verde | Raso | S12 | 13.779 | | -17.378 |
| Canary | M.Clara | P3 | | 12.634 | | -16.379 | | C.Verde | Raso | S12 | 14.116 | | -15.867 |
| Canary | M.Clara | P3 | | 11.901 | | -16.507 | | C.Verde | Raso | S12 | 12.630 | | -17.658 |
| Canary | M.Clara | P3 | | 11.201 | | -16.623 | | C.Verde | Cima | P1 | 13.122 | | -16.913 |
| Canary | M.Clara | P3 | | 12.699 | | -16.119 | | C.Verde | Cima | P1 | 12.211 | | -16.502 |
| Canary | M.Clara | P3 | | 11.557 | | -16.686 | | C.Verde | Cima | P1 | 11.895 | | -16.741 |
| Canary | M.Clara | P3 | | 12.105 | | -16.388 | | C.Verde | Cima | P1 | 12.103 | | -17.256 |
| Canary | M.Clara | P5 | | 12.776 | | -16.276 | | C.Verde | Cima | P3 | 13.369 | | -17.167 |
| Canary | M.Clara | P5 | | 13.140 | | -16.513 | | C.Verde | Cima | P3 | 11.881 | | -16.539 |
| Canary | M.Clara | P5 | | 12.785 | | -15.792 | | C.Verde | Cima | P3 | 11.889 | | -17.075 |
| Canary | M.Clara | P5 | | 12.563 | | -16.475 | | C.Verde | Cima | P3 | 11.842 | | -17.395 |
| Canary | M.Clara | P5 | | 11.768 | | -16.735 | | C.Verde | Cima | P5 | 12.197 | | -16.935 |
| Canary | M.Clara | P5 | | 12.385 | | -16.296 | | C.Verde | Cima | P5 | 11.476 | | -16.353 |
| Canary | M.Clara | P5 | | 12.394 | | -16.218 | | C.Verde | Cima | P5 | 12.066 | | -16.023 |
| Canary | M.Clara | P7 | | 14.768 | | -16.780 | | C.Verde | Cima | P5 | 11.938 | | -17.416 |
| Canary | M.Clara | P7 | | 13.343 | | -16.806 | | C.Verde | Cima | P7 | 12.526 | | -16.478 |
| Canary | M.Clara | P7 | | 14.597 | | -16.319 | | C.Verde | Cima | P7 | 12.905 | | -16.222 |
| Canary | M.Clara | P7 | | 13.517 | | -16.734 | | C.Verde | Cima | P7 | 12.075 | | -16.835 |
| Canary | M.Clara | P7 | | 12.686 | | -16.483 | | C.Verde | Cima | P7 | 13.851 | | -17.435 |
| Canary | M.Clara | P7 | | 12.206 | | -16.558 | | C.Verde | Cima | P10 | 12.999 | | -16.538 |
| Canary | M.Clara | P7 | | 15.435 | | -17.137 | | C.Verde | Cima | P10 | 12.084 | | -16.437 |
| Canary | M.Clara | P7 | | 13.102 | | -16.138 | | C.Verde | Cima | P10 | 11.973 | | -16.386 |
| Canary | M.Clara | P10 | | 15.179 | | -17.814 | | C.Verde | Cima | P10 | 14.460 | | -17.414 |
| Canary | M.Clara | P10 | | 12.170 | | -16.465 | | C.Verde | Cima | S1 | 12.686 | | -16.676 |
| Canary | M.Clara | P10 | | 14.695 | | -16.263 | | C.Verde | Cima | S1 | 12.398 | | -16.345 |
| Canary | M.Clara | P10 | | 13.618 | | -16.488 | | C.Verde | Cima | S1 | 11.895 | | -16.385 |
| Canary | M.Clara | P10 | | 14.114 | | -17.323 | | C.Verde | Cima | S1 | 11.570 | | -17.783 |
| Canary | M.Clara | P10 | | 12.649 | | -16.207 | | C.Verde | Cima | S8 | 12.741 | | -16.353 |
| Canary | M.Clara | P10 | | 15.635 | | -16.804 | | C.Verde | Cima | S8 | 12.666 | | -16.051 |
| Canary | M.Clara | P10 | | 12.735 | | -16.317 | | C.Verde | Cima | S8 | 11.976 | | -16.579 |
| Canary | M.Clara | S1 | | 13.469 | | -16.336 | | C.Verde | Cima | S8 | 14.351 | | -16.928 |
| Canary | M.Clara | S1 | | 14.299 | | -16.328 | | C.Verde | Cima | S12 | 12.433 | | -16.196 |
| Canary | M.Clara | S1 | | 13.326 | | -16.550 | | C.Verde | Cima | S12 | 12.021 | | -16.299 |
| Canary | M.Clara | S1 | | 11.566 | | -16.622 | | C.Verde | Cima | S12 | 11.874 | | -16.042 |
| Canary | M.Clara | S1 | | 12.270 | | -16.836 | | C.Verde | Cima | S12 | 11.888 | | -17.133 |
| Canary | M.Clara | S1 | | 15.097 | | -17.042 | |  |  |  |  | |  |

Information about the breeding areas (populations and islets), feathers sampled [the 1^st^ primary (P1), 3^rd^ (P3), 5^th^ (P5), 7^th^ (P7) and 10^th^ (P10)], the 1^st^ secondary (S1), 8^th^ (S8) and 12^th^ (S12)], and δ^15^N and δ^13^C values of the feathers.

**Table B.** Isotopic data of tracked Bulwer’s petrels and complementary information.

| **Breeding area** | | | | **GLS information** | | **Non-breeding area** | | | **P1** | | **S8** | | **R6** | |
| --- | --- | --- | --- | --- | --- | --- | --- | --- | --- | --- | --- | --- | --- | --- |
| **Population** | **Islet** | **Lon** | **Lat** | **Year**  **recovery** | **ID** | **Lon** | **Lat** | **Cluster** | **δ^15^N** | **δ ^13^C** | **δ ^15^N** | **δ ^13^C** | **δ ^15^N** | **δ ^13^C** |
| Azores | Vila | -25.17 | 36.94 | 2008 | 6082001_1 | -20.20 | -25.72 | S.Atlantic | 12.340 | -16.361 | 14.306 | -16.862 | 11.896 | -17.098 |
| Azores | Vila | -25.17 | 36.94 | 2008 | 6085001_1 | -17.85 | -1.81 | C.Atlantic | 11.861 | -16.276 | 14.246 | -16.099 | 15.767 | -16.588 |
| Azores | Vila | -25.17 | 36.94 | 2008 | 6086001_1 | -12.43 | -19.85 | S.Atlantic | 11.823 | -16.229 | 14.238 | -16.352 | 15.057 | -16.645 |
| Azores | Vila | -25.17 | 36.94 | 2008 | 6094001_1 | -32.73 | 2.90 | C.Atlantic | 11.815 | -16.577 | 12.571 | -15.998 | 11.671 | -16.212 |
| Azores | Vila | -25.17 | 36.94 | 2008 | 6096001_1 | -12.84 | -27.99 | S.Atlantic | 11.668 | -15.800 | 14.738 | -16.949 | 17.119 | -16.847 |
| Azores | Vila | -25.17 | 36.94 | 2008 | 6098001_1 | -28.05 | -25.15 | S.Atlantic | 11.333 | -15.846 | 12.957 | -16.385 | 11.263 | -17.241 |
| Azores | Vila | -25.17 | 36.94 | 2008 | 6099001_1 | -33.37 | 7.27 | C.Atlantic | 12.270 | -16.403 | 13.266 | -16.116 | 13.628 | -16.074 |
| Canary | M.Clara | -13.53 | 29.29 | 2011 | 17245001_1 | -27.22 | 1.94 | C.Atlantic | 12.027 | -16.539 | 14.251 | -16.226 | 16.208 | -17.492 |
| Canary | M.Clara | -13.53 | 29.29 | 2011 | 17246001_1 | -8.83 | -26.25 | S.Atlantic | 12.263 | -16.352 | 14.415 | -16.516 | 15.965 | -17.691 |
| Canary | M.Clara | -13.53 | 29.29 | 2011 | 17251001_1 | -3.58 | -25.07 | S.Atlantic | 12.317 | -16.430 | 16.626 | -17.194 | 16.329 | -17.470 |
| Canary | M.Clara | -13.53 | 29.29 | 2011 | 17253001_1 | -4.10 | -26.39 | S.Atlantic | 13.238 | -16.339 | 16.053 | -16.981 | 15.934 | -17.918 |
| Canary | M.Clara | -13.53 | 29.29 | 2011 | 17254001_1 | -35.39 | 9.21 | C.Atlantic | 11.278 | -16.578 | 12.545 | -15.874 | 12.128 | -16.514 |
| Canary | M.Clara | -13.53 | 29.29 | 2011 | 17255001_1 | -26.88 | 12.84 | C.Atlantic | 11.987 | -16.266 | 12.046 | -16.242 | 11.849 | -16.463 |
| Canary | M.Clara | -13.53 | 29.29 | 2011 | 17256001_1 | -33.72 | -23.13 | S.Atlantic | 12.048 | -16.651 | 14.011 | -16.705 | 11.889 | -17.225 |
| Canary | M.Clara | -13.53 | 29.29 | 2011 | 17257001_1 | -29.45 | 13.78 | C.Atlantic | 12.727 | -16.698 | 13.739 | -16.325 | 11.343 | -16.117 |
| Canary | M.Clara | -13.53 | 29.29 | 2011 | 17258001_1 | -33.90 | 7.23 | C.Atlantic | 12.166 | -16.609 | 13.655 | -16.078 | 13.415 | -16.193 |
| Canary | M.Clara | -13.53 | 29.29 | 2011 | 17259001_1 | -29.15 | 10.50 | C.Atlantic | 13.117 | -16.863 | 12.558 | -16.027 | 13.820 | -16.878 |
| Canary | M.Clara | -13.53 | 29.29 | 2011 | 17260001_1 | -28.81 | 11.20 | C.Atlantic | 10.812 | -16.247 | 11.983 | -16.273 | 12.394 | -16.457 |
| Canary | M.Clara | -13.53 | 29.29 | 2011 | 17262001_1 | -13.00 | -26.73 | S.Atlantic | 13.278 | -16.297 | 15.619 | -17.242 | 15.558 | -17.749 |
| Canary | M.Clara | -13.53 | 29.29 | 2011 | 17263001_1 | -8.24 | -27.46 | S.Atlantic | 10.995 | -16.990 | 14.734 | -16.989 | 16.012 | -18.187 |
| Canary | M.Clara | -13.53 | 29.29 | 2011 | 17264001_1 | -7.97 | -24.87 | S.Atlantic | 12.045 | -16.294 | 15.142 | -16.975 | 15.937 | -17.836 |
| Canary | M.Clara | -13.53 | 29.29 | 2012 | 18B423001_1 | -24.82 | 12.44 | C.Atlantic | 12.036 | -15.991 | 12.937 | -16.057 | 12.280 | -16.137 |
| Canary | M.Clara | -13.53 | 29.29 | 2012 | 18B425001_1 | -3.90 | -24.12 | S.Atlantic | 13.982 | -16.757 | 16.793 | -17.330 | 16.465 | -17.746 |
| Canary | M.Clara | -13.53 | 29.29 | 2012 | 18B427001_1 | -26.83 | 14.51 | C.Atlantic | 13.020 | -16.418 | 13.323 | -16.431 | 12.182 | -16.433 |
| Canary | M.Clara | -13.53 | 29.29 | 2012 | 18B429001_1 | -29.63 | 10.56 | C.Atlantic | 13.164 | -16.334 | 12.232 | -15.711 | 11.734 | -16.160 |
| Canary | M.Clara | -13.53 | 29.29 | 2012 | 18B430001_1 | -7.16 | -25.10 | S.Atlantic | 12.634 | -16.516 | 16.132 | -17.130 | 15.618 | -17.996 |
| Canary | M.Clara | -13.53 | 29.29 | 2012 | 18B432001_1 | -33.42 | -1.38 | C.Atlantic | 12.799 | -16.267 | 13.073 | -16.184 | 13.085 | -16.649 |
| Canary | M.Clara | -13.53 | 29.29 | 2012 | 18B433001_1 | -28.59 | 13.82 | C.Atlantic | 12.373 | -16.349 | 12.579 | -15.884 | 11.294 | -16.350 |
| Canary | M.Clara | -13.53 | 29.29 | 2012 | 18B435001_1 | -31.19 | 11.54 | C.Atlantic | 12.896 | -16.364 | 12.553 | -15.850 | 12.417 | -16.110 |
| Canary | M.Clara | -13.53 | 29.29 | 2012 | 18B439001_1 | -20.82 | -9.27 | S.Atlantic | 12.968 | -16.707 | 15.330 | -16.314 | 14.902 | -16.909 |
| Canary | M.Clara | -13.53 | 29.29 | 2012 | 18B440001_1 | -25.07 | 12.83 | C.Atlantic | 12.863 | -16.556 | 13.358 | -16.367 | 12.077 | -16.366 |
| Canary | M.Clara | -13.53 | 29.29 | 2012 | 18B446001_1 | -28.63 | -2.31 | C.Atlantic | 12.918 | -16.645 | 13.586 | -16.398 | 15.732 | -17.886 |
| Canary | M.Clara | -13.53 | 29.29 | 2012 | 18B449001_1 | -35.55 | 4.98 | C.Atlantic | 12.232 | -16.483 | 14.130 | -16.219 | 12.994 | -16.077 |
| Canary | M.Clara | -13.53 | 29.29 | 2012 | 18B451001_1 | -0.76 | -25.76 | S.Atlantic | 12.866 | -16.573 | 16.479 | -17.087 | 16.605 | -17.076 |
| Canary | M.Clara | -13.53 | 29.29 | 2013 | 13141004_1 | -4.72 | -30.13 | S.Atlantic | 11.965 | -16.848 | 15.589 | -16.793 | 16.826 | -18.030 |
| Canary | M.Clara | -13.53 | 29.29 | 2013 | 17265001_3 | -27.84 | 24.10 | C.Atlantic | 12.505 | -16.841 | 10.892 | -17.126 | 10.953 | -17.625 |
| Canary | M.Clara | -13.53 | 29.29 | 2013 | 18B022001_1 | -23.98 | -15.52 | S.Atlantic | 12.598 | -16.905 | 15.356 | -16.725 | 14.250 | -16.522 |
| Canary | M.Clara | -13.53 | 29.29 | 2013 | 18B030001_1 | -4.09 | -27.03 | S.Atlantic | 13.148 | -17.088 | 15.219 | -16.441 | 16.288 | -17.414 |
| Canary | M.Clara | -13.53 | 29.29 | 2013 | 18B077001_1 | -30.68 | 11.89 | C.Atlantic | 13.096 | -16.847 | 13.973 | -16.266 | 13.805 | -16.366 |
| Canary | M.Clara | -13.53 | 29.29 | 2013 | 18B425002_1 | -8.93 | -21.41 | S.Atlantic | 13.114 | -16.526 | 15.003 | -16.673 | 16.081 | -17.788 |
| Canary | M.Clara | -13.53 | 29.29 | 2013 | 18B440002_1 | -26.77 | 12.72 | C.Atlantic | 12.948 | -17.094 | 13.024 | -16.354 | 12.488 | -16.521 |
| Canary | M.Clara | -13.53 | 29.29 | 2013 | 18B445001_2 | -20.18 | -26.87 | S.Atlantic | 13.124 | -16.826 | 14.404 | -17.249 | 13.938 | -16.779 |
| Canary | M.Clara | -13.53 | 29.29 | 2013 | V395008001_1 | -12.72 | -9.43 | S.Atlantic | 12.763 | -17.181 | 15.282 | -16.690 | 16.997 | -17.398 |
| Canary | M.Clara | -13.53 | 29.29 | 2013 | V395010001_1 | -22.78 | -11.71 | S.Atlantic | 13.125 | -16.724 | 14.777 | -16.968 | 13.982 | -16.424 |
| Canary | M.Clara | -13.53 | 29.29 | 2013 | V395011001_1 | -23.87 | 17.81 | C.Atlantic | 13.559 | -16.600 | 13.187 | -16.861 | 12.536 | -16.816 |
| Canary | M.Clara | -13.53 | 29.29 | 2013 | V395015001_1 | -35.49 | -21.81 | S.Atlantic | 12.436 | -16.688 | 10.414 | -17.069 | 11.518 | -17.061 |
| Canary | M.Clara | -13.53 | 29.29 | 2013 | V395016001_1 | -27.91 | 13.08 | C.Atlantic | 13.031 | -17.127 | 14.430 | -17.146 | 12.330 | -16.552 |
| Canary | M.Clara | -13.53 | 29.29 | 2013 | V395017001_1 | -27.44 | 12.42 | C.Atlantic | 12.397 | -16.748 | 12.399 | -16.386 | 12.893 | -16.308 |
| Canary | M.Clara | -13.53 | 29.29 | 2014 | 17835001_1 | -30.16 | 11.08 | C.Atlantic | 13.381 | -16.603 | 11.990 | -16.353 | 13.136 | -16.591 |
| Canary | M.Clara | -13.53 | 29.29 | 2014 | 18B427003_3 | -24.29 | -1.42 | C.Atlantic | 12.578 | -16.731 | 13.527 | -16.378 | 14.259 | -16.857 |
| Canary | M.Clara | -13.53 | 29.29 | 2014 | 18B434001_3 | -29.17 | -4.07 | C.Atlantic | 14.148 | -16.808 | 13.212 | -17.595 | 13.543 | -17.984 |
| Canary | M.Clara | -13.53 | 29.29 | 2014 | 18B436003_1 | -24.50 | -26.49 | S.Atlantic | 12.662 | -16.307 | 13.982 | -17.114 | 13.798 | -17.740 |
| C.Verde | Raso | -24.60 | 16.61 | 2008 | 6101001_1 | -22.68 | -5.57 | C.Atlantic | 11.836 | -16.446 | 14.156 | -16.217 | 13.439 | -15.913 |
| C.Verde | Raso | -24.60 | 16.61 | 2008 | 6230001_1 | -36.25 | 3.39 | C.Atlantic | 11.501 | -15.973 | 14.343 | -16.137 | 14.135 | -16.345 |
| C.Verde | Raso | -24.60 | 16.61 | 2008 | 6232001_1 | -25.64 | 5.96 | C.Atlantic | 12.343 | -16.701 | 12.275 | -16.299 | 11.633 | -17.047 |
| C.Verde | Raso | -24.60 | 16.61 | 2008 | 6234001_1 | -13.04 | -12.85 | S.Atlantic | 11.777 | -16.269 | 14.782 | -16.361 | 15.147 | -16.510 |
| C.Verde | Raso | -24.60 | 16.61 | 2008 | 6235001_1 | -26.16 | 10.34 | C.Atlantic | 11.650 | -16.600 | 11.378 | -16.533 | 11.057 | -16.259 |
| C.Verde | Raso | -24.60 | 16.61 | 2008 | 6237001_1 | -22.62 | -1.77 | C.Atlantic | 11.779 | -16.436 | 12.968 | -16.097 | 12.694 | -16.474 |
| C.Verde | Raso | -24.60 | 16.61 | 2008 | 6238001_1 | -35.22 | 10.06 | C.Atlantic | 12.295 | -16.512 | 13.455 | -16.546 | 12.115 | -16.216 |
| C.Verde | Raso | -24.60 | 16.61 | 2009 | 6230002_1 | -23.83 | -9.39 | S.Atlantic | 12.004 | -16.461 | 14.096 | -16.455 | 13.812 | -16.307 |
| C.Verde | Raso | -24.60 | 16.61 | 2009 | 6233001_2 | -7.29 | -9.45 | S.Atlantic | 12.825 | -16.391 | 12.719 | -16.442 | 14.975 | -16.946 |
| C.Verde | Raso | -24.60 | 16.61 | 2009 | 6940001_1 | -25.46 | 14.18 | C.Atlantic | 12.437 | -16.750 | 12.934 | -16.373 | 11.717 | -16.934 |
| C.Verde | Raso | -24.60 | 16.61 | 2009 | 6945001_1 | -21.51 | -2.23 | C.Atlantic | 12.926 | -16.169 | 13.833 | -15.594 | 13.906 | -16.566 |
| C.Verde | Raso | -24.60 | 16.61 | 2010 | 13192001_1 | -36.59 | 15.93 | C.Atlantic | 12.012 | -16.271 | 13.190 | -16.165 | 12.203 | -16.249 |
| C.Verde | Raso | -24.60 | 16.61 | 2010 | 13193001_1 | -24.43 | 19.00 | C.Atlantic | 12.790 | -16.424 | 12.830 | -16.458 | 12.171 | -16.698 |
| C.Verde | Raso | -24.60 | 16.61 | 2010 | 6948001_2 | -21.17 | 2.50 | C.Atlantic | 12.761 | -16.445 | 13.938 | -16.386 | 14.586 | -16.423 |
| C.Verde | Raso | -24.60 | 16.61 | 2010 | 8339002_1 | -23.63 | -2.85 | C.Atlantic | 13.059 | -16.501 | 14.176 | -16.479 | 14.428 | -16.407 |
| C.Verde | Cima | -24.64 | 14.97 | 2011 | 17252001_1 | -32.36 | 6.48 | C.Atlantic | 13.088 | -16.663 | 13.271 | -16.258 | 12.480 | -16.370 |
| C.Verde | Cima | -24.64 | 14.97 | 2011 | 17270001_1 | -32.33 | 3.51 | C.Atlantic | 12.578 | -16.239 | 13.441 | -16.281 | 13.463 | -16.381 |
| C.Verde | Cima | -24.64 | 14.97 | 2011 | 17274001_1 | -29.89 | 12.27 | C.Atlantic | 12.124 | -16.497 | 12.564 | -16.041 | 11.864 | -16.450 |
| C.Verde | Cima | -24.64 | 14.97 | 2011 | 17275001_1 | -31.14 | 10.48 | C.Atlantic | 12.574 | -16.739 | 13.563 | -16.581 | 11.959 | -16.259 |
| C.Verde | Cima | -24.64 | 14.97 | 2011 | 17281001_1 | -12.96 | -5.12 | S.Atlantic | 13.212 | -16.741 | 13.403 | -16.281 | 14.542 | -16.597 |
| C.Verde | Cima | -24.64 | 14.97 | 2012 | 17042002_1 | -12.78 | -17.63 | S.Atlantic | 12.810 | -16.782 | 13.205 | -16.275 | 15.430 | -17.123 |
| C.Verde | Cima | -24.64 | 14.97 | 2012 | 17045002_1 | -18.68 | 1.70 | C.Atlantic | 12.997 | -17.174 | 14.627 | -16.258 | 12.463 | -16.410 |
| C.Verde | Cima | -24.64 | 14.97 | 2012 | 17046002_1 | -27.87 | 13.51 | C.Atlantic | 12.063 | -16.281 | 11.671 | -16.139 | 12.424 | -16.106 |
| C.Verde | Cima | -24.64 | 14.97 | 2012 | 17245002_1 | -41.32 | 8.98 | C.Atlantic | 12.204 | -16.873 | 12.372 | -16.206 | 12.261 | -16.453 |
| C.Verde | Cima | -24.64 | 14.97 | 2012 | 17251002_1 | -35.14 | 6.37 | C.Atlantic | 12.085 | -16.588 | 13.187 | -16.079 | 11.902 | -16.503 |
| C.Verde | Cima | -24.64 | 14.97 | 2012 | 17254002_1 | -17.71 | -6.87 | S.Atlantic | 12.868 | -16.094 | 13.246 | -16.396 | 15.237 | -16.786 |
| C.Verde | Cima | -24.64 | 14.97 | 2012 | 17256002_1 | -30.35 | 13.05 | C.Atlantic | 12.307 | -16.961 | 13.311 | -16.353 | 12.346 | -16.544 |
| C.Verde | Cima | -24.64 | 14.97 | 2012 | 17258002_1 | -24.89 | 10.13 | C.Atlantic | 12.541 | -16.403 | 12.491 | -16.484 | 11.762 | -16.616 |
| C.Verde | Cima | -24.64 | 14.97 | 2012 | 17260002_1 | -24.26 | 0.30 | C.Atlantic | 12.552 | -16.827 | 12.383 | -16.372 | 13.026 | -16.413 |
| C.Verde | Cima | -24.64 | 14.97 | 2012 | 17263002_1 | -27.13 | -1.48 | C.Atlantic | 12.894 | -16.464 | 13.650 | -16.489 | 13.037 | -16.646 |
| C.Verde | Cima | -24.64 | 14.97 | 2012 | 17269001_2 | -36.14 | 6.56 | C.Atlantic | 12.005 | -16.532 | 12.605 | -15.994 | 12.331 | -16.872 |
| C.Verde | Cima | -24.64 | 14.97 | 2012 | 17847001_1 | -25.20 | -0.13 | C.Atlantic | 13.046 | -16.533 | 12.829 | -16.605 | 15.171 | -16.971 |
| C.Verde | Cima | -24.64 | 14.97 | 2012 | 17848001_1 | -33.94 | 5.40 | C.Atlantic | 11.862 | -16.073 | 13.159 | -16.080 | 12.027 | -15.971 |
| C.Verde | Cima | -24.64 | 14.97 | 2012 | 17849001_1 | -33.86 | 5.26 | C.Atlantic | 12.931 | -16.287 | 13.136 | -16.365 | 12.501 | -16.436 |

Information about the breeding areas [populations, islets, longitud (Lon) and latitud (Lat)], information about the GLS (years of recovery and IDs), information about the non-breeding areas [longitud (Lon) and latitud (Lat) of the centroids during the non-breeding seasson, and the belonging to a cluster [Central (C.Atlantic) or South Atlantic (S.Atlantic)], and δ^15^N and δ^13^C values for the 1^st^ primary (P1), 8^th^ secondary (S8) and 6^th^ rectrice (R6) feathers of Bulwer’s petrel sampled at GLS recovery. The clasters were calculated applying a partition around medoids algorithm to an orthometric distance matrix created among all the centroids for the non-breeding areas of the last year recorded by the GLS, and we determined a priori the number of clusters as the number that maximized the *overall average silhouette width* criterion.
